# Dietary novel alkaline protease from *Bacillus licheniformis* improves broiler meat nutritional value and modulates intestinal microbiota and metabolites

**DOI:** 10.1186/s42523-023-00287-z

**Published:** 2024-01-06

**Authors:** Wuzhou Yi, Yanjie Liu, Shijun Fu, Jianshu Zhuo, Jiping Wang, Tizhong Shan

**Affiliations:** 1 College of Animal Sciences, Zhejiang University, Hangzhou, China; 2 The Key Laboratory of Molecular Animal Nutrition, Ministry of Education, Hangzhou, China; 3 Zhejiang Provincial Laboratory of Feed and Animal Nutrition, Hangzhou, China; 4 Shandong Binzhou Animal Science and Veterinary Medicine Academy, Binzhou, China; 5 Jinan Bestzyme Bio-Engineering Co., Ltd, Jinan, China; 6 Wellhope Foods Co., Ltd, Jinan, China

**Keywords:** Inosine monophosphate, Fatty acid, Amino acid, Gut microbiota

## Abstract

**Background:**

Different types of exogenous protease supplements have a positive impact on animal performance, but their effects on the nutritional value of meat and the gut microbial community of broilers have not been extensively studied. The objective of this investigation was to determine the impact of supplementation with a novel alkaline protease derived from *Bacillus licheniformis* (at doses of 0, 100, 200, 300, and 400 g/t) on the fatty acid and amino acid profiles, inosine monophosphate (IMP) levels, total volatile basic nitrogen (TVB-N) content found within the breast muscle, as well as the impact on the cecal microbiota and metabolites.

**Results:**

Supplementation with 200–400 g/t of the novel protease resulted in a significant elevation in the concentration of essential amino acids (*P* < 0.001), flavor amino acids (*P* < 0.001), and total protein (*P* = 0.013) within the breast muscle. Results derived from the 16S rRNA sequencing and untargeted metabolomics analysis of the cecal content revealed that the novel protease reshaped the cecal microbial and metabolite profiles. In particular, it led to increased relative abundances of *Bacteroides*, *Lactobacillus*, *Alistipes*, and *Eubacterium*, while simultaneously causing a reduction in the metabolites of D-lactic acid and malonic acid. Moreover, correlation analyses unveiled significant relationships between distinct microbes and metabolites with the contents of IMP, fatty acids, and amino acids in the broiler's breast muscle.

**Conclusion:**

In summary, the novel protease regulated the intestinal microbial community and metabolism, thereby inducing changes in the compositions of fatty acids and amino acids profiles, as well as IMP levels in broiler meat. These alterations significantly contributed to the enhancement of the nutritional value and flavor of the meat.

**Graphical abstract:**

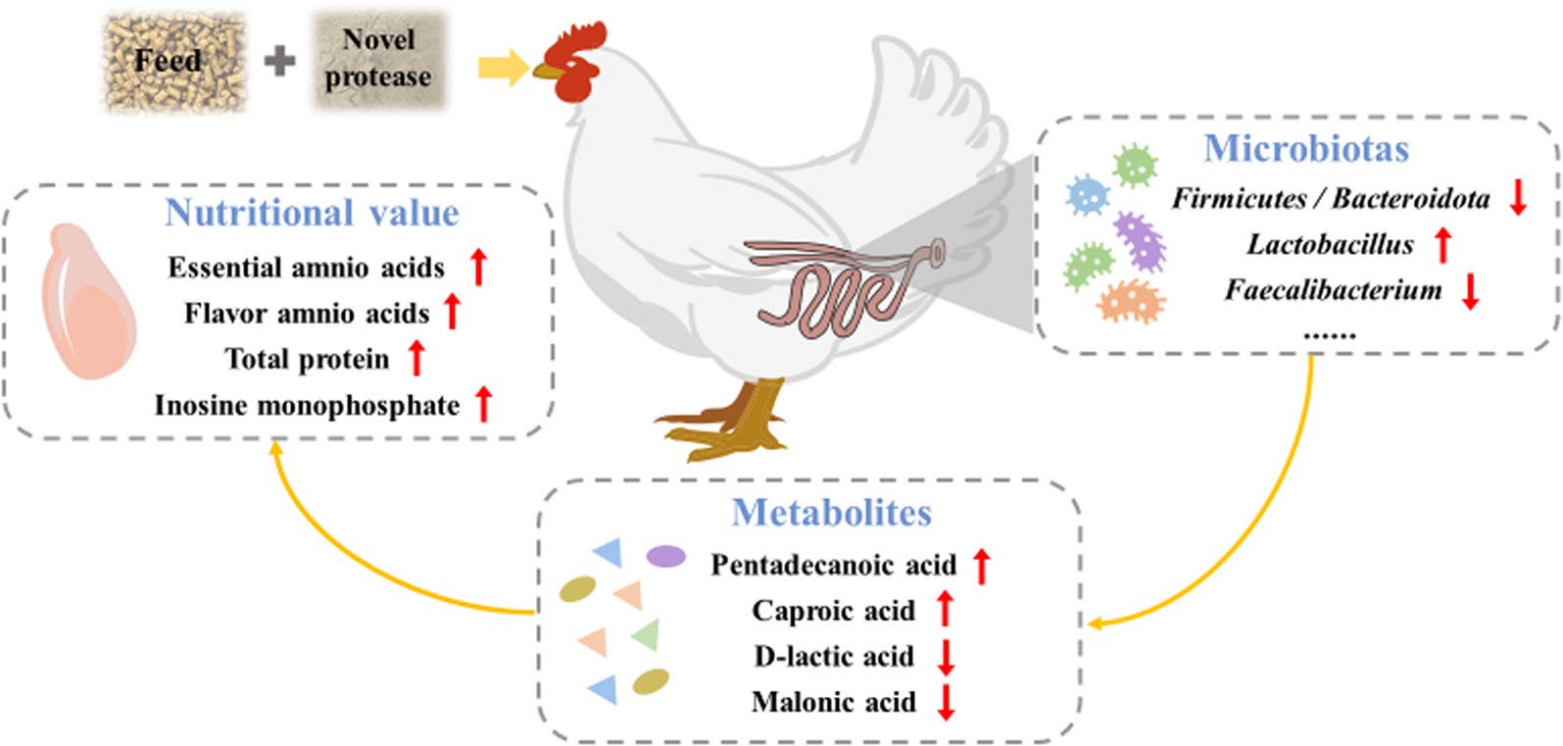

**Supplementary Information:**

The online version contains supplementary material available at 10.1186/s42523-023-00287-z.

## Background

In recent decades, the global landscape of poultry production and consumption has been marked by a consistent uptrend, making poultry one of the most widely consumed meat worldwide [1]. Broiler meat, a prime source of superior quality animal protein, has gained increasing the acceptability of broiler meat among consumers, elevated protein content, coupled with low cholesterol, calorie, and fat levels [2]. Consumer acceptance of broiler meat depends on factors such as its color, flavor, tenderness, juiciness, and nutritional composition [3]. The composition of amino acids and fatty acids in meat reflects its nutritional value and flavor. Flavor amino acids (FAA), including glutamic, aspartic, alanine, arginine, and glycine, play a significant role in meat flavor. Among these, alanine, serine, and glycine undergo chemical reactions during heating, resulting in a sweet taste in meat [4]. Additionally, unsaturated fatty acids (UFA) in meat, such as n-3 PUFA and n-6 PUFA, play a crucial role in determining meat quality [5, 6]. These fatty acids have beneficial effects on human health, including the prevention of cardiovascular diseases [7] and the improvement of non-alcoholic fatty liver disease [8]. Moreover, inosine monophosphate (IMP) is a nucleotide that contributes to the intense umami taste in meat, especially when combined with sodium glutamate [4, 9]. It is considered a crucial indicator for assessing the flavor and acceptability of meat products. The term TVB-N refers to volatile substances, including ammonia and amines, that are produced during protein degradation in animal food, and it serves as an important indicator of food freshness [3].

The supplementation of exogenous enzymes in the feed of livestock can improve the digestion efficiency thereby improving the performance of animals [10]. However, not all proteases have demonstrated favorable production outcomes. Some studies suggested that the growth performance and protein utilization of broilers were not significantly affected by the addition of protease [11]. These results can be attributed to the inadequate coordination between added exogenous protease and the endogenous protease. Moreover, limited research has been conducted to investigate whether the addition of protease can alter the nutritional value of meat and gut microbiota in broilers. Thus, we developed a novel alkaline protease derived from a thermophilic bacterium, *Bacillus licheniformis*. And in this study, we mainly evaluated this novel protease effect on nutritional value, flavor and freshness of meat and gut microbiota in broiler. Meanwhile, limited research has been conducted to investigate whether the addition of protease can alter the fatty acid and amino acid profiles of broiler meat. As a result, there is a lack of comprehensive understanding of the effects of this novel protease on broilers. Therefore, the objective of this study was to assess the impact of incorporating the novel protease on the flavor, fatty acid composition, and amino acid profile of broiler meat. The hypothesis for this experiment was that the addition of the novel protease could modulate the colonization of intestinal microbes, alter intestinal metabolites, enhance the deposition of nutrient substance in broiler meat, and ultimately improve the nutritional value of meat. This experimental investigation is expected to provide essential theoretical support for the application of protease supplementation to alter the flavor, fatty acid composition, and amino acid profiles of broiler meat.

## Results

### Growth performance and antioxidant capacity of breast muscle.

The body weight increased significantly (*P* < 0.05) with increasing levels of novel protease in the corn-soybean meal diet (Additional file 1: Fig. S1). Furthermore, malondialdehyde (MDA) content significantly decreased (*P* < 0.05), and the activity of total antioxidant capacity (T-AOC) increased (*P* < 0.05) with the addition of novel protease. These results indicate that protease supplementation may have benefits on body weight and antioxidant activities in broilers.

### Muscle amino acids profile

The addition of novel protease to the diet of broilers resulted in significant changes in the composition of amino acids present in their breast muscle when compared to the control group (Table 1). The content of flavor amino acids (linear, *P* < 0.001; quadratic, *P* < 0.001), essential amino acids (linear, *P* < 0.001; quadratic, *P* < 0.001), total amino acids (linear, *P* < 0.001; quadratic, *P* < 0.001) and total protein (linear, *P* = 0.003; quadratic, *P* = 0.013) increased significantly with increasing levels of novel protease in the corn-soybean meal diet. The content of tyrosine (linear, *P* = 0.026; quadratic, *P* = 0.063) decreased markedly with increasing levels of novel protease supplementation. Furthermore, the content of aspartic acid, threonine, serine, glutamic, glycine, alanine, valine, methionine, isoleucine, leucine, phenylalanine, lysine, histidine and arginine, with the exception of proline, also exhibited a significant increase (*P* < 0.05) with the addition of novel protease.

**Table 1:** Effects of novel protease on total protein and amino acids profiles in breast muscle of broilers

Items	Protease (g/t)					SEM	*P* value	L	Q
	0	100	200	300	400		A		
Aspartic, g/100g	1.943^b^	1.942^b^	2.020^a^	2.080^a^	2.063^a^	0.014	< 0.001	< 0.001	< 0.001
Threonine, g/100g	0.940^b^	0.937^b^	0.990^a^	0.978^a^	0.957^ab^	0.006	0.015	0.093	0.032
Serine, g/100g	0.755^b^	0.780^ab^	0.790^ab^	0.815^a^	0.807^a^	0.006	0.014	0.001	0.002
Glutamic, g/100g	3.045^c^	3.110^bc^	3.228^a^	3.285^a^	3.210^ab^	0.023	0.001	0.001	< 0.001
Proline, g/100g	0.767	0.782	0.798	0.772	0.772	0.008	0.750	1.000	0.552
Glycine, g/100g	0.925^bc^	0.908^c^	0.952^abc^	0.995^a^	0.975^ab^	0.009	0.009	0.002	0.011
Alanine, g/100g	1.208^b^	1.187^b^	1.230^b^	1.312^a^	1.287^a^	0.011	< 0.001	< 0.001	< 0.001
Valine, g/100g	1.110^b^	1.077^b^	1.132^b^	1.197^a^	1.188^a^	0.011	< 0.001	< 0.001	0.001
Methionine, g/100g	0.592^bc^	0.585^c^	0.615^ab^	0.637^a^	0.630^a^	0.005	0.001	< 0.001	0.001
Isoleucine, g/100g	1.048^bc^	1.023^b^	1.087^ab^	1.108^a^	1.095^a^	0.008	0.002	0.002	0.007
Leucine, g/100g	1.710^b^	1.682^b^	1.785^a^	1.843^a^	1.830^a^	0.015	< 0.001	< 0.001	< 0.001
Tyrosine, g/100g	0.965^a^	0.763^c^	0.898^ab^	0.782^bc^	0.778^bc^	0.023	0.007	0.026	0.063
Phenylalanine, g/100g	0.882^c^	1.040^b^	1.018^b^	1.208^a^	1.197^a^	0.027	< 0.001	< 0.001	< 0.001
Lysine, g/100g	1.922^b^	1.895^b^	2.030^a^	2.012^a^	2.008^a^	0.014	< 0.001	0.001	0.003
Histidine, g/100g	0.743^ab^	0.682^b^	0.727^ab^	0.798^a^	0.798^a^	0.013	0.006	0.009	0.010
Arginine, g/100g	1.372^b^	1.377^b^	1.452^a^	1.445^a^	1.428^a^	0.009	< 0.001	0.001	0.001
EAA, g/100g	7.612^c^	7.643^c^	8.042^b^	8.357^a^	8.275^ab^	0.067	< 0.001	< 0.001	< 0.001
FAA, g/100g	8.968^c^	8.922^c^	9.347^b^	9.690^a^	9.510^ab^	0.069	< 0.001	< 0.001	< 0.001
TAA, g/100g	19.933^b^	19.767^b^	20.750^a^	21.283^a^	21.033^a^	0.145	< 0.001	< 0.001	< 0.001
TP, g/100g	21.350^bc^	21.200^c^	21.550^bc^	22.817^a^	22.450^ab^	0.192	0.013	0.003	0.013

Results are the means of each group of 6 broilers. EAA, essential amino acid; FAA, flavour amnio acid; TAA, total amino acid; TP, total protein; A, ANOVA; L, linear; Q, quadratic

^a^^−^^c^Means within a row with different superscripts are significantly different (*P* < 0.05)

### Muscle fatty acids profile

The results presented in Table 2 indicate that the addition of novel protease to the broiler diet at increasing concentrations changed the levels of certain fatty acids in broiler breast muscle. These include myristic acid (C14:0) (linear, *P* = 0.024; quadratic, *P* = 0.077), palmitic acid (C16:0) (linear,* P* = 0.045; quadratic, *P* = 0.134), palmitoleic acid (C16:1) (linear, *P* = 0.036; quadratic, *P* = 0.115), oleic acid (C18:1 n9) (linear, *P* = 0.016; quadratic, *P* = 0.058), and eicosenoic acid (C20:1) (linear, *P* = 0.021; quadratic, *P* = 0.069). The ratio of PUFA to SFA significantly decreased (*P* = 0.009) with the supplementation of 200 g/t novel protease. Specifically, the levels of SFA, PUFA, n-3 PUFA, n-6 PUFA, total fatty acid content (TFAC), and intramuscular fat (IMF) were found to be the lowest in the group receiving a protease addition at a dosage of 300 g/t.

**Table 2:** Effects of novel protease on fat content and fatty acid profiles in breast muscle of broilers

Items	Protease (g/t)					SEM	*P* value	L	Q
	0	100	200	300	400		A		
C8:0, g/100g	0.0106^b^	0.0114^b^	0.0214^a^	0.0090^b^	0.0087^b^	0.001	0.001	0.482	0.031
C14:0, g/100g	0.0116	0.0101	0.0099	0.0077	0.0081	0.001	0.238	0.024	0.077
C16:0, g/100g	0.4728	0.4640	0.4670	0.3385	0.3692	0.024	0.220	0.045	0.134
C16:1, g/100g	0.0764	0.0736	0.0601	0.0542	0.0462	0.005	0.368	0.036	0.115
C18:0, g/100g	0.1775	0.1862	0.2210	0.1355	0.1717	0.010	0.077	0.371	0.492
C18:1n9, g/100g	0.6265	0.6270	0.5577	0.4248	0.4287	0.036	0.177	0.016	0.058
C18:2n6, g/100g	0.3305	0.3377	0.3173	0.2367	0.2657	0.018	0.313	0.068	0.194
C18:3n3, g/100g	0.0147	0.0152	0.0133	0.0099	0.0105	0.001	0.291	0.041	0.127
C20:1, g/100g	0.0078	0.0077	0.0067	0.0054	0.0060	< 0.001	0.170	0.021	0.069
C20:2, g/100g	0.0081	0.0081	0.0073	0.0070	0.0087	< 0.001	0.741	0.953	0.566
C20:3n6, g/100g	0.0154	0.0171	0.0148	0.0134	0.0182	0.001	0.366	0.753	0.550
C22:1n9, g/100g	0.0071^b^	0.0111^b^	0.0161^a^	0.0080^b^	0.0109^b^	0.001	< 0.001	0.423	0.043
C22:6n3, g/100g	0.0060	0.0058	0.0066	0.0053	0.0076	< 0.001	0.379	0.296	0.407
SFA, g/100g	0.6725	0.6717	0.7193	0.4907	0.5577	0.033	0.165	0.082	0.198
MUFA, g/100g	0.7178	0.7193	0.6406	0.4924	0.4917	0.042	0.196	0.018	0.063
PUFA, g/100g	0.3747	0.3838	0.3593	0.2722	0.3107	0.020	0.349	0.087	0.238
n-3 PUFA, g/100g	0.0207	0.0210	0.0199	0.0152	0.0181	0.001	0.510	0.182	0.412
n-6 PUFA, g/100g	0.3459	0.3547	0.3321	0.2500	0.2839	0.018	0.330	0.079	0.219
PUFA: SFA, g/100g	0.5589^a^	0.5675^a^	0.4910^b^	0.5568^a^	0.5536^a^	0.009	0.047	0.749	0.300
n-6: n-3, g/100g	16.7626	17.0661	16.6527	16.3823	15.8423	0.224	0.519	0.112	0.204
TFAC, g/100g	1.77	1.78	1.73	1.25	1.36	0.094	0.223	0.042	0.126
IMF, g/100g	1.93	1.95	1.95	1.37	1.52	0.105	0.223	0.055	0.153

Results are the means of each group of 6 broilers. SFA, saturated fatty acids; MUFA, monounsaturated fatty acids; PUFA, polyunsaturated fatty acids; TFAC, total fatty acids; IMF, intramuscular fat; A, ANOVA; L, linear; Q, quadratic

^a^^−^^b^Means within a row with different superscripts are significantly different (*P* < 0.05)

### The content of IMP and TVB-N values

The content of IMP is a crucial indicator of the umami taste in meat. According to the results displayed in Table 3, the content of IMP in the muscle of broilers significantly increased (linear, *P* = 0.001; quadratic, *P* = 0.003) with an increasing concentration of novel protease added to the diet. Also presented in Table 3, supplementation of novel protease in the diet led to a decrease in the TVB-N value of breast muscle refrigerated at 4 °C at 0 days (linear, *P* = 0.005; quadratic, *P* = 0.016) and 7 days (linear, *P* = 0.004; quadratic, *P* = 0.017). The addition of 300 g/t and 400 g/t of novel protease significantly decreased (*P* = 0.023) the TVB-N value at 7 days. This suggests that the protease supplementation improved the flavor and freshness of the broiler meat by reducing the level of TVB-N.

**Table 3:** Effects of novel protease on flavor and freshness value in breast muscle of broilers

Items	Protease (g/t)					SEM	*P* value	L	Q
	0	100	200	300	400		A		
IMP, ppm	1765.29^c^	1856.56^bc^	2122.70^ab^	2170.83^ab^	2195.86^a^	53.814	0.017	0.001	0.003
TVB-N, g/100g									
0d	9.69^ab^	9.82^a^	9.66^ab^	9.27^c^	9.35^bc^	0.064	0.017	0.005	0.016
3d	10.68	10.47	10.91	10.74	10.53	0.258	0.987	0.994	0.963
5d	12.03	12.16	12.30	11.43	11.06	0.472	0.922	0.433	0.653
7d	22.71^b^	24.37^a^	20.81^bc^	19.99^c^	19.38^c^	0.575	0.023	0.004	0.017
9d	29.36	29.42	27.80	28.28	29.43	0.795	0.954	0.863	0.811

Results are the means of each group of 6 broilers. IMP, inosine monophosphate; TVB-N, total volatile basic nitrogen; A, ANOVA; L, linear; Q, quadratic

^a^^−^^c^Means within a row with different superscripts are significantly different (*P* < 0.05)

### Intestinal microbiota

#### Beta and alpha diversity

**Fig. 1:**
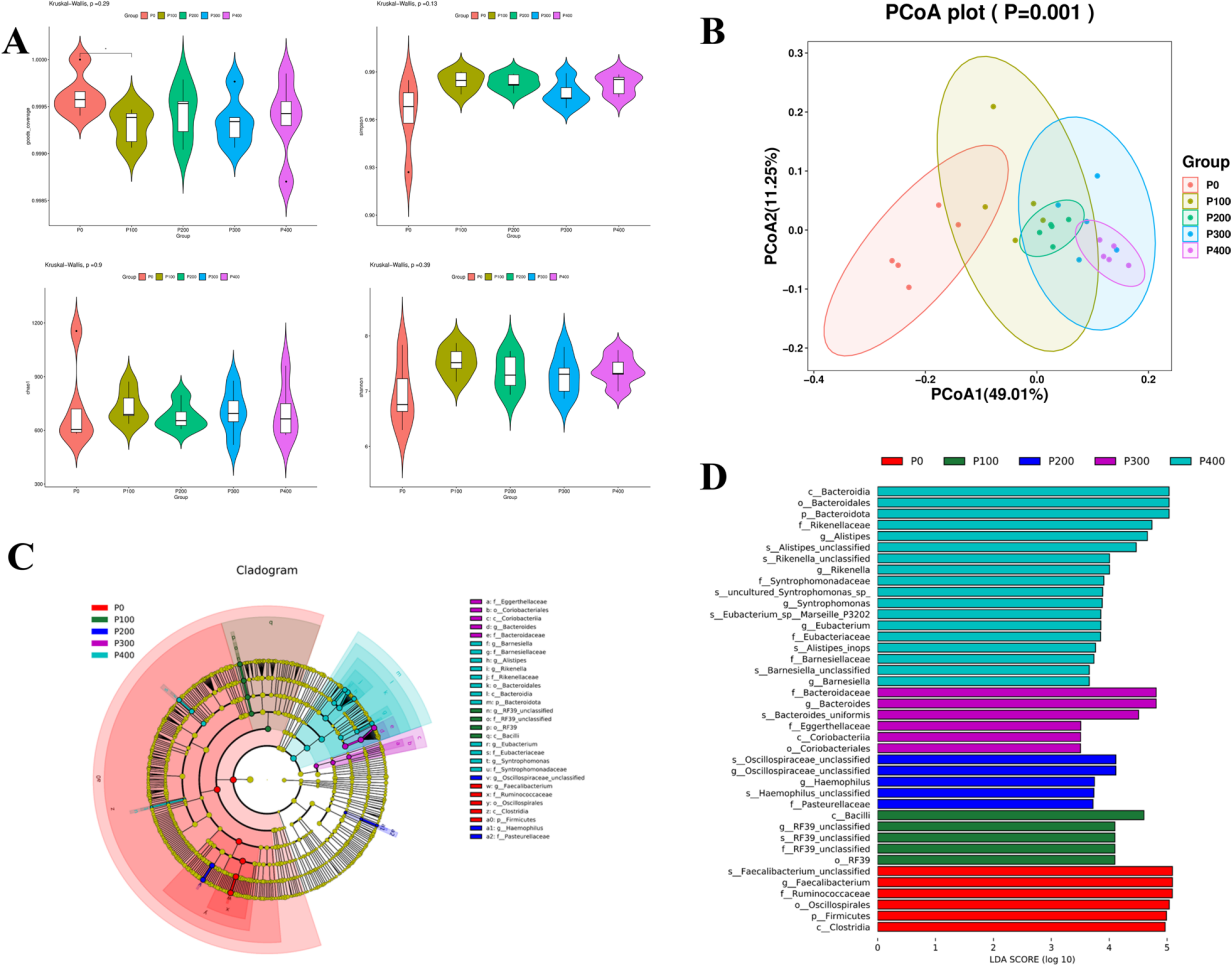
Diversity analyses of microbial communities among groups. **A** Alpha diversity (goods coverage, Simpson, chao1, and Shannon). **B** Principal coordinates analysis (PCoA) of microbial communities among groups based on Bray–Curtis distance. **C** The cladogram of Linear discriminant analysis (LDA) effect size (LEfSe) analysis. **D** The histogram of LEfSe analysis. p_, phylum level; c_, class level; o_, order level; f_, family level; g_, genus level; s_, species. (n = 5 per group)

The results of this study suggest that the supplementation of novel protease had a significant effect on the diversity of the intestinal microbiota of broilers. Specifically, the microbial abundance (Goods Coverage) in the group supplemented with 100 g/t of protease was significantly lower than the control group (*P* < 0.05) (Fig. 1A). However, no significant differences were observed in the α diversity (the Simpson, Chao1, and Shannon indices) of the cecum among the other groups (*P* > 0.05). The Principal Coordinate Analysis (PCoA) based on the Bray–Curtis distance, which is a measure of dissimilarity between different microbial communities, showed that the microbial community structure was significantly influenced by the protease treatment. This was evident from the formation of two distinct clusters: one formed by the control group, and the other by the groups supplemented with 100–400 g/t of protease (Fig. 1B).

#### LEfSe analysis

The linear discriminant analysis Effect Size (LEfSe) analysis further highlighted significant differences in the relative abundance of bacteria in the cecal microbiota among the five groups. In the context of LEfSe analysis, bacterial taxa with LDA scores greater than 3.5 were selected as biomarker taxa. The analysis revealed 40 taxa biomarkers across the four experimental groups (Fig. 1C, D). These biomarkers mainly belonged to the *Bacteroidia* class, the *Bacteroidales* order, and the *Bacteroidaceae* and *Rikenellaceae* families, as well as the *Alistipes* and *Bacteroides* genera.

#### ANOVA for different microbes

**Fig. 2:**
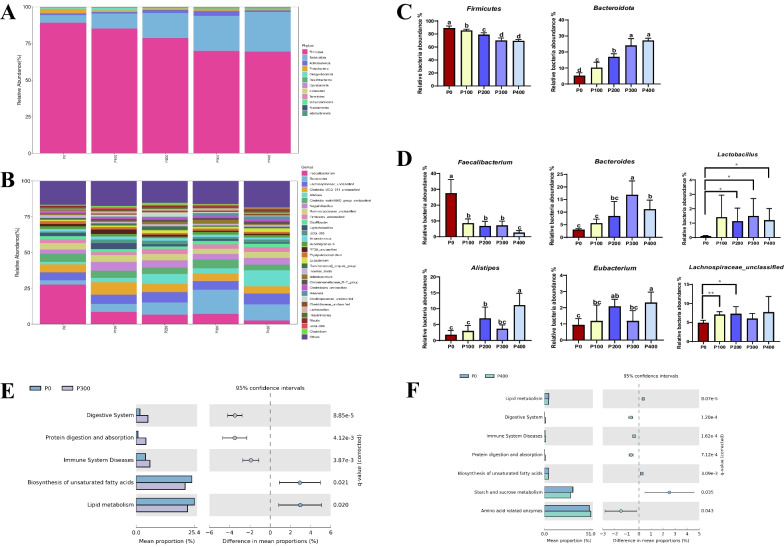
Average relative abundance of microbial species in the cecum at the phylum level (**A**) and genus level (**B**). Relative abundance of microbial communities in the cecum contents at the phylum level (**C**) and genus level (**D**). Comparison of predicted metabolic pathway abundances between the groups by STAMP. **E** P0 versus P300; **F** P0 versus P400. ^a−c^Different lowercase letters indicate significant difference between groups (*P* < 0.05). Confidence Interval was set at 95%

The relative abundances of different bacterial phyla and genera are displayed in Fig. 2A, B. Further analysis revealed that as the supplementation of the novel protease increased, there was a gradual decrease in the relative abundance of *Firmicutes* at the phylum level, while the relative abundance of *Bacteroidota* increased accordingly (Fig. 2C). At the genus level, supplementation of the diet with 100 g/t of the novel protease led to a significant increase in the abundance of *Lachnospiraceae unclassified* (*P* < 0.01), while it decreased the abundance of *Faecalibacterium* (*P* < 0.05). The relative abundance of *Bacteroides* and *Lactobacillus* significantly increased (*P* < 0.05) with 300 and 400 g/t protease supplementation, which also led to a decrease in *Faecalibacterium* (*P* < 0.05). Supplementation at 400 g/t additionally resulted in significant increases in the abundance of *Alistipes* (*P* < 0.05) and *Eubacterium* (*P* < 0.05) (Fig. 2D).

#### Differential metabolite KEGG analysis

The PICRUSt2 was employed to infer the metabolic functions predicted for the intestinal microbiota. Further analysis based on level 3 of the microbial-predicted pathway functions using the STAMP confirmed differences in metabolic functions (Fig. 2E, F). Notably, both 300 and 400 g/t supplementation levels significantly enhanced 3 metabolic pathways (digestive system, protein digestion and absorption, and immune system diseases; *P* < 0.05), while reducing the biosynthesis of unsaturated fatty acids and lipid metabolism. This group also demonstrated a significant increase in enzymes related to amino acid metabolism (*P* < 0.05).

### Metabolomics analysis

**Fig. 3:**
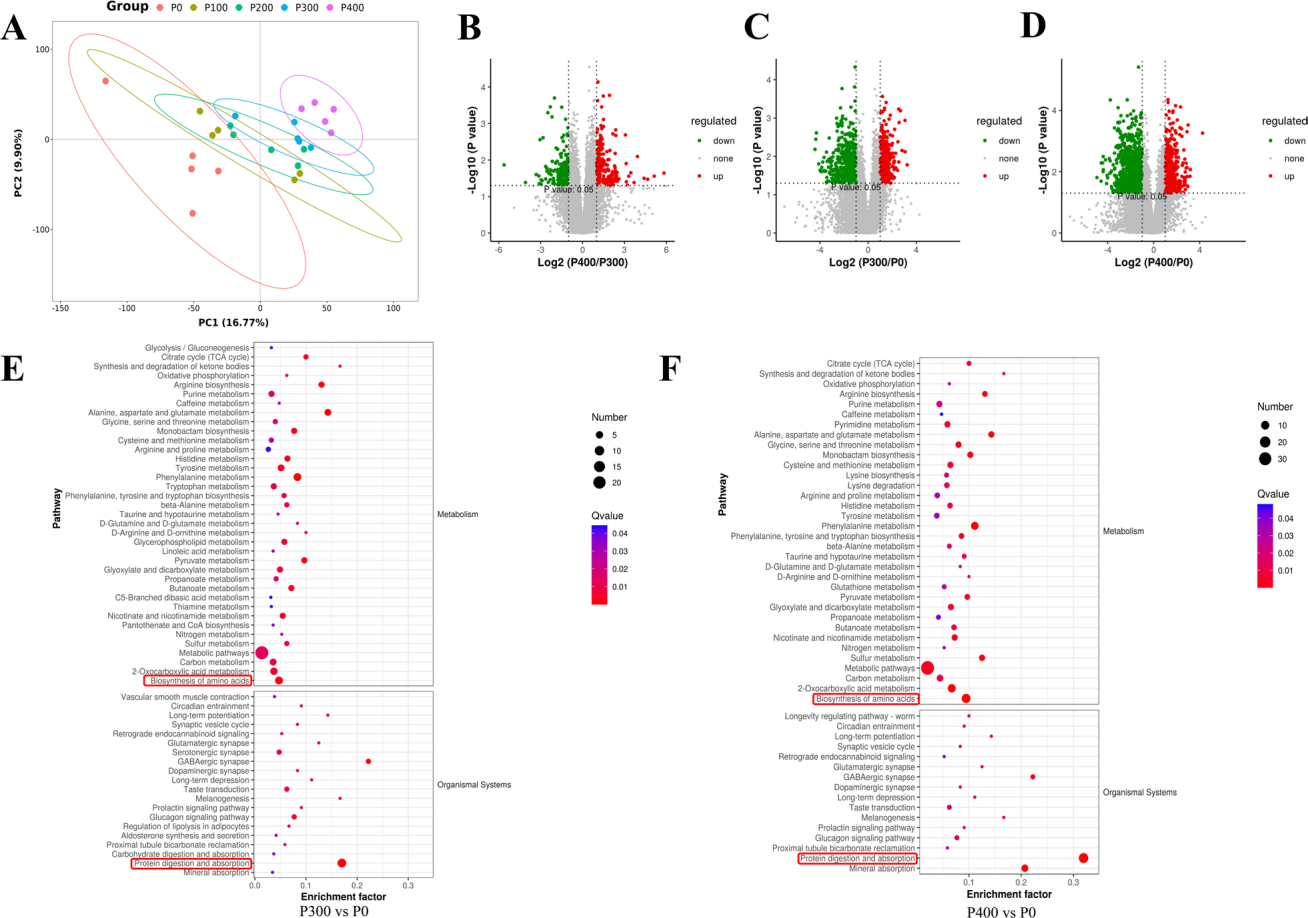
Cecal content non-targeted metabolite dataset. **A** Partial least squares discriminant analysis (PLS-DA) of metabolite composition in broilers after novel protease treatment. **B**–**D** Changes in cecal metabolites among P400, P300 and P0 groups. **E**, **F** Pathway enrichment analysis was performed using the significantly different metabolites between P400 versus P0 and P300 versus P0

To elucidate the impact of protease supplementation on the metabolic profiles and pathways within the intestinal microbiome, untargeted metabolomics analyses were performed on cecal content samples from all broiler groups. Meat quality-related phenotypic indices, such as IMP, amino acid, and fatty acid profiles, showed the most significant variations between the P300 and P400 groups compared to the P0 group. The investigation initially centered on identifying the common differential metabolites in the P300 and P400 groups. Subsequently, the analysis was extended to assess the variations of these metabolites in the remaining two groups. The PLS-DA (Partial Least Squares Discriminant Analysis) plot shown in Fig. 3A, indicates a clear separation between the cecal contents samples of the P0 group and those of the P300 group, as well as between the P0 group and the P400 group. However, there is little separation evident between the P300 group and the P400 group. These findings suggest that protease supplementation has a significant impact on the metabolic profiles in the cecum, resulting in different metabolic profiles between the protease groups and the control group. However, the metabolic profiles of the groups receiving 300 g/t and 400 g/t protease supplementation were quite similar, suggesting a certain degree of metabolic commonality at these higher supplementation levels.

**Fig. 4:**
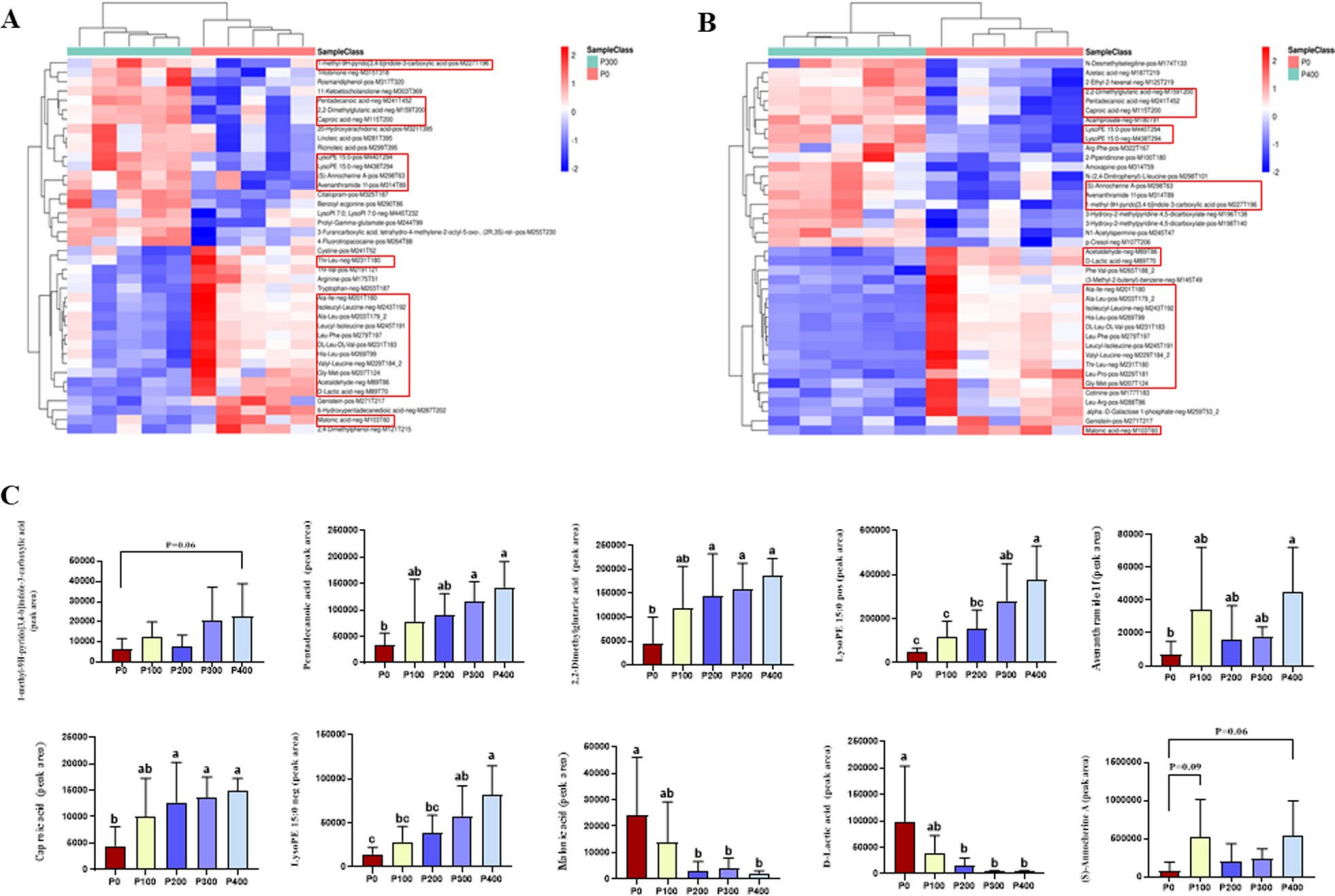
Changed metabolites in cecum contents. **A**, **B** Heatmap of top 40 differentially expressed metabolites between P400 versus P0 and P300 versus P0. **C** Changes in major metabolites in cecal contents. ^a−c^Different lowercase letters indicate significant difference between groups (*P* < 0.05)

Our analyses further identified 975 metabolites in the cecal contents that displayed differential expression between the P300 and P0 groups. Of these metabolites, 387 were upregulated, while 588 were downregulated in the P300 group in comparison to the P0 group (Fig. 3B). Similarly, when comparing the P400 group and the P0 group, a total of 1716 differentially expressed metabolites were detected, which included 1073 downregulated metabolites and 643 upregulated metabolites in the P400 group (Fig. 3C). In the comparison of the P400 group and the P300 group, 388 differentially expressed metabolites were identified, of which 182 were downregulated and 206 were upregulated in the P400 group (Fig. 3D). In order to elucidate the biological implications of the differentially expressed metabolites, we undertook KEGG pathway enrichment analysis. The mentioned metabolites, differentially expressed within the cecal contents following the application of a novel protease (300 and 400 g/t), demonstrated a significant enrichment in select metabolic pathways when compared to the control group. These metabolic pathways notably comprised amino acid biosynthesis, and protein digestion and absorption (Fig. 3E, F). The heatmap results, as shown in Fig. 4A, B, display the differential metabolite contents between the novel protease groups (300 and 400 g/t) and the control group. In particular, some differential metabolites such as pentadecanoic acid, 2,2-dimethylglutaric acid, caproic acid, lysoPE 15:0 pos and lysoPE 15:0 neg were found to be significantly increased (*P* < 0.05) in the groups treated with novel protease (300 and 400 g/t). However, the contents of D-lactic acid and malonic acid were noticeably decreased (*P* < 0.05) in the groups receiving novel protease supplementation at both the 300 and 400 g/t levels (Fig. 4C).

### Correlation of significantly different microbial species, metabolites and meat nutritional value

**Fig. 5:**
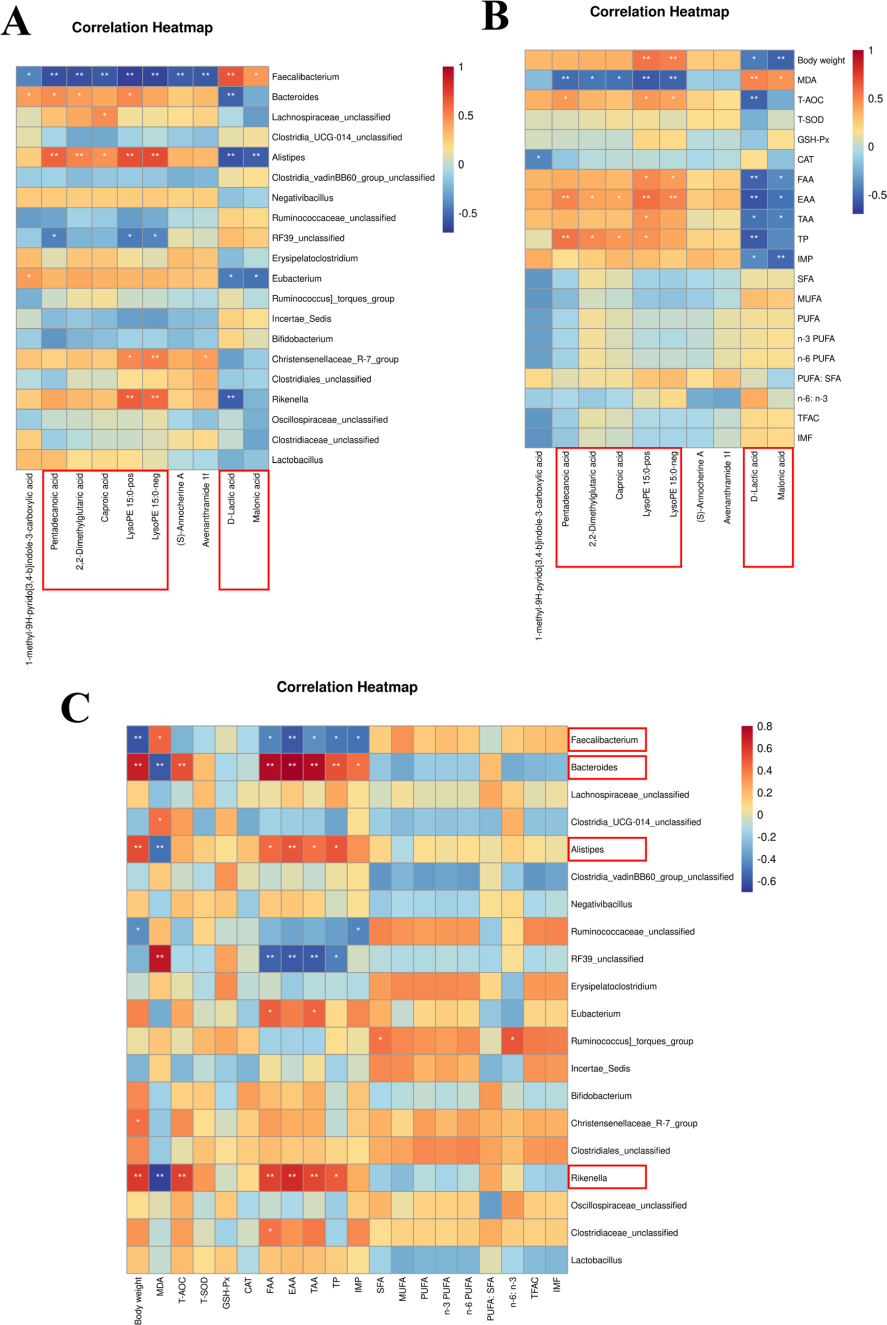
Correlations of significantly different microbial species at genus level, metabolites and meat nutritional values (amino acids, fatty acids, IMP, and antioxidant capacity). The correlation effect is indicated by a color gradient from blue (negative correlation) to red (positive correlation) *, *P* < 0.05; **, *P* < 0.01

In order to investigate the conceivable interconnections among the gut microbiota, metabolites, and meat nutritional value parameters (inclusive of amino acids, fatty acids, IMP, and antioxidant capacity), we constructed correlation matrices employing the Spearman correlation method. The relationships between the differentially expressed metabolites, meat nutritional value metrics, and microbiota at the genus level within the cecal contents subsequent to the novel protease regimen (ranging from 100 to 400 g/t) across both the treatment groups and the control group are illustrated in Fig. 5.

The Spearman correlation depicted in Fig. 5A reveals that the upregulated metabolites in the cecal contents from the group treated with the novel protease, encompassing compounds such as pentadecanioc acid, 2,2-dimethylglutaric acid, caproic acid, lysoPE 15:0 pos, and lysoPE 15:0 neg, demonstrated a positive correlation with *Alistipes* (*P* < 0.05), and a negative correlation with *Faecalibacterium* (*P* < 0.01). Moreover, lysoPE 15:0 pos and lysoPE 15:0 neg showed positively correlated with *Rikenella* (*P* < 0.01) and *Christensenellaceae R-7 group* (*P* < 0.05), and negatively correlated with *RF39 unclassified* (*P* < 0.05). Conversely, the downregulated metabolites from the protease-treated group, including D-lactic acid and malonic acid, showed a positive correlation with *Faecalibacterium* (*P* < 0.05), and a negative correlation with *Alistipes* (*P* < 0.05) and *Eubacterium* (*P* < 0.05). In additioin, D-lactic acid showed negatively correlated with *Rikenella* (*P* < 0.01) and *Bacteroides* (*P* < 0.01).

As illustrated in Fig. 5B, the five metabolites that were significantly upregulated in the novel protease treatment groups exhibited a positive correlation with EAA (*P* < 0.05), and a negative correlation with MDA (*P* < 0.05). But the content of TP in breast muscle showed a positive correlation with these metabolites (*P* < 0.05) except lysoPE 15:0 neg. And lysoPE 15:0 pos and lysoPE 15:0 neg showed positively correlated with T-AOC (*P* < 0.05), body weight (*P* < 0.01) and FAA (*P* < 0.05). The two significantly downregulated metabolites, D-lactic acid and malonic acid, were positively correlated with MDA (*P* < 0.05), yet demonstrated a negative correlation with IMP (*P* < 0.05), FAA (*P* < 0.05), TAA (*P* < 0.05), EAA (*P* < 0.05), and body weight (*P* < 0.05). And D-lactic acid showed negatively correlated with TP (*P* < 0.01) and T-AOC (*P* < 0.01).

Additionally, as indicated by the Spearman correlation in Fig. 5C, *Bacteroides*, *Alistipes* and *Rikenella* were found to have positive associations with TP (*P* < 0.05), EAA (*P* < 0.01), FAA (*P* < 0.05), TAA (*P* < 0.05), T-AOC (*P* < 0.01) and body weight (*P* < 0.01). These bacteria exhibited a negative correlation with MDA (*P* < 0.01). *Faecalibacterium* showed a positive correlation with MDA (*P* < 0.05), yet displayed a significant negative association with TP (*P* < 0.05), body weight (*P* < 0.01), EAA (*P* < 0.01), FAA (*P* < 0.05), TAA (*P* < 0.05), and IMP (*P* < 0.05). Pertaining to the key indicator used to evaluate meat flavor, IMP was positively associated with *Bacteroides* (*P* < 0.05) and negatively correlated with *Faecalibacterium* (*P* < 0.05) and *Ruminococcaceae unclassified* (*P* < 0.05).

## Discussion

The constitution of amino acids and fatty acids in animal musculature serves as a critical determinant of meat nutritional value [12, 13]. Our experimental results suggested that dietary novel protease led to a reduction in the levels of myristic acid (C14:0) (linear, *P* = 0.024; quadratic, *P* = 0.077) and palmitic acid (C16:0) (linear, *P* = 0.045; quadratic, *P* = 0.134), which aligns with other researches. A study reported a substantial decline in these acids in broiler breast fed a complex enzyme preparation (xylanase + amylase + protease) [14]. Given that current nutritional guidelines advocate for a reduction in SFA intake, particularly myristic acid (14:0) and palmitic acid (16:0), which have been associated with heightened low density lipoprotein (LDL) cholesterol levels and negative impacts on human health [15]. FAA, encompassing glycine, aspartic acid, alanine, phenylalanine, glutamic acid, and tyrosine, play an indispensable role in determining the freshness of food [16, 17]. Our evidence demonstrated that dietary protease enhanced the content of FAA in the muscle, thereby amplifying the flavor of broiler meat and making it more palatable to consumers. Additionally, in the breast muscle, both EAA and TAA showed an increase due to protease supplementation, which corroborates with other studies [16]. The EAA augmentation in broilers fed a protease-supplemented diet intimates that protease supplementation may potentiate the protein quality and nutritive value of the muscle.

IMP is a purine nucleotide intricately involved in nucleotide metabolism. Furthermore, IMP serves as a flavor-enhancing agent, imparting desirable qualities such as flavor, tenderness, juiciness, and umami character to broiler meat [18, 19]. Previous investigations have suggested that the elevation in IMP content is primarily attributed to the increased creatine content and bolstered fatty acid oxidation. Our experimental observations underscored a significant elevation in the content of creatine, accompanied by an increase in IMP levels. The rudimentary precursors necessitated in the de novo synthesis pathway of IMP encompass a single carbon unit from N^10^-formyl-THF, phosphoribose, amino acids, and CO_2_ [19]. A number of genes are associated with the synthesis and metabolism of IMP, such as *IMP cyclohydrolase*, *glutamine-PRPP amidotransferase, adenylosuccinate lyase and adenosine monophosphate deaminase 1* [19]. The potential impact of the novel protease on genes associated with IMP synthesis or transport in the breast or intestine warrants additional investigation.

TVB-N represents a volatile compound generated through protein degradation in animal-based foods, attributable to enzymatic and bacterial activity. It primarily encompasses ammonia, amines, and other basic nitrogenous substances. Hence, the extent of amino acid degradation in food exhibits a positive correlation with TVB-N content, making TVB-N an integral indicator for assessing meat freshness in accordance with national standards [3]. TVB-N can exert deleterious impacts on the tyrosine and methionine constituents in food, culminating in the diminution of nutritional value and potentially compromising human health [20]. In the current investigation, our data demonstrated that the TVB-N levels exhibited a decline with the novel protease supplementation at both 0 (*P* = 0.017) and 7 days (*P* = 0.023), indicative of protease's potential to prolong shelf life and augment meat nutritional value.

Many studies have established that protease can impact the composition of the intestinal microbiota in broilers [21, 22]. Our investigation corroborates prior research in identifying *Firmicutes* and *Bacteroidetes* as the predominant phyla in the cecum of broilers [23, 24]. The *Firmicutes*/*Bacteroidetes* ratio is frequently mentioned in scientific literature as an obesity biomarker, with obese animals typically showing increased *Firmicutes* abundance and decreased *Bacteroidetes* [25, 26]. We found that protease supplementation led to a reduction in *Firmicutes* and an increase in *Bacteroidetes*, which implies potential fat accumulation reduction in broilers and enhanced protein deposition in the breast muscle. *Bacteroidetes* is known to produce several beneficial gut metabolites, including tryptamine [27], propionate [28], vitamins B [29] all of which contribute to intestinal health and barrier integrity maintenance. Our data demonstrated an increased *Bacteroides* abundance with the addition of novel protease compared to the control diet. We also noticed a significant elevation in the abundance of *Eubacterium* and *Alistipes*, two crucial intestinal bacteria that produce short-chain fatty acids, essential for maintaining host health and safeguarding the intestinal mucosal barrier [30, 31]. The findings from our study revealed a notable increase in *Lactobacillus* abundance following the administration of 200–400 g/t of a novel protease supplementation. This aligns with prior research indicating that dietary *Bacillus subtilis*, used as an antibiotic alternative, substantially enhanced *Lactobacillus* presence in the cecum [32]. Notably, *Lactobacillus*, functioning as a probiotic, along with combined probiotics and Yeast, has been shown to potentially substitute synthetic antibiotics in enhancing broiler growth performance and carcass traits [33]. It is hypothesized that *Lactobacillus* can produce lactic acid, which may lower intestinal pH and exerts a strong inhibitory effect on other genera in an acidic environment [34]. Therefore, a higher *Lactobacillus* species proportion allows its members to occupy a wide gut niche and ultimately plays a crucial role in protecting unsaturated fatty acids from oxidation, acting as an efficient antioxidant [35]. Furthermore, we noted that dietary novel protease at doses of 100 g/t and 200 g/t substantially increased the abundance of *Lachnospiraceae unclassified*. *Lachnospiraceae* is known for their health-promoting abilities, including the production of host nutrients, energy supply to the colonic epithelium, and the maintenance of host immune homeostasis [36].

To understand how novel proteases can enhance broiler breast muscle quality by regulating broiler physiological metabolism, we analyzed the cecal contents of broilers using metabolomics. Our findings showed that novel protease supplementation significantly modified the cecal metabolic profiles. Pentadecanoic acid, an odd-chain saturated fatty acid (OCFA), has been linked in studies to higher circulating levels associated with lower obesity risks [37, 38], cardiovascular disease [39], metabolic syndrome [40], type 2 diabetes [41, 42], and other diseases. Lower pentadecanoic acid dietary intake and blood concentrations are associated with higher mortality and a deteriorated physiological state [43]. Caproic acid, a short chain fatty acids (SCFA), is generated by gut microbes fermenting dietary fiber and resistant starch, contributing to the gut microbiota's beneficial effects and promoting host and gut microbiota health [44]. Phospholipids, with their broad biological functions energy storage, signal transduction promotion, and cell membrane structural integrity maintenance are important. Many distinct phospholipids can be cleaved by specific phospholipases, with lysoPE produced from structural phospholipids' hydrolysis by phospholipase A2 [45]. LysoPE, a naturally occurring lipid, has regulatory effects in senescence and ripening [46]. Lactic acid exists as two enantiomers, with L-lactic acid being a common metabolic compound, while certain microbial strains or some unrelated metabolic pathways produce D-lactic acid. D-lactic acid, the harmful enantiomer, can be generated through multiple pathways, including food contamination and microbiota during certain pathological states, such as intestinal injury [47]. Malonic acid, a three carbon compound, acts as a competitive inhibitor of succinate dehydrogenase in many microorganisms, potentially inhibiting the TCA cycle and cell growth [48]. Our study revealed that compared to the control group, novel protease supplementation in the cecum increased levels of pentadecanoic acid, caproic acid, and lysoPE and decreased levels of D-lactic acid and malonic acid. These results suggest that novel protease supplementation increases beneficial metabolites and reduces adverse metabolites in broilers' cecum, thus effectively improving broiler cecal metabolism. Furthermore, according to KEGG pathway enrichment analysis, we found that the addition of novel proteases enhanced the expression of amino acid synthesis and protein digestion and absorption pathways. We also demonstrated that these up-regulated metabolites were positively associated with *Alistipes*, *Bacteroide*s, and *Firmicutes unclassified*, but negatively correlated with *Faecalibacterium* and *RF39 unclassified*. Studies have reported a negative correlation between *Bacteroides* and obesity [49]. Moreover, *Bacteroides* and *Alistipes* are the primary producers of SCFA [50].

The gut microbiota and its metabolites are intimately linked to the quality of broiler meat and the profiles of amino acids and fatty acids within it [51–54]. However, the effects of the novel protease on gut microbiota and metabolites and the potential correlation between differential microbiota, amino acids, and fatty acids in meat remain unclear. Our results demonstrated that *Bacteroides* were positively associated with numerous indicators of growth performance, amino acid and antioxidant capacity in breast. *Bacteroides* have been reported to participate in various significant metabolic activities, including carbohydrate fermentation, nitrogenous substance utilization, and bile acids and other steroids' biotransformation [55]. Our findings suggest that *Bacteroides* are positively associated with EAA, TAA and FAA. However, another study found that *Bacteroides* were negatively correlated with amino acid composition in broiler breast muscle [52]. These inconsistent findings suggest that the relationship between gut microbiota and changes in amino acids of meat needs more investigation to be verified. The *Rikenellaceae* family, a recently established family in the *Bacteroidales* order, currently includes two genera: *Alistipes* and *Rikenella*. These anaerobic gut bacteria can produce SCFA by fermenting indigestible dietary fibers in the gut [56, 57]. In our study, *Alistipes* and *Rikenella* exhibited a robust positive correlation with caproic acid, confirming that novel protease enhance SCFA production in the gut. Previous studies have shown that SCFA can act as a critical energy source for intestinal epithelial cells, regulate intestinal blood flow, and stimulate enterocyte growth and proliferation [58]. SCFA production contributes to the gut microbiota's beneficial effects and supports the health of the host and the intestinal microbial community [44]. These findings indicate that novel protease enhances growth performance and antioxidant capacity, and these changes are associated with the production and functions of differential bacteria and SCFA in intestinal tract.

## Conclusions

Our study provides evidence suggesting that the novel protease improves the nutritional value and flavor of meat by altering the profiles of amino acids and IMP. Furthermore, the protease restructures the gut microbial community, resulting in an increase in SCFA-producing bacteria such as *Eubacterium*, *Alistipes*, and *Rikenella*. Additionally, treatment with the novel protease enhances the content of metabolites, such as pentadecanoic acid and caproic acid, which are beneficial to animal health. The observed correlations suggest that the novel protease modulates the intestinal microbial community and metabolism, consequently altering the composition of amino acid profiles and IMP in breast muscle. However, the specific mechanisms underlying these effects warrant further investigation.

## Materials and methods

### Ethics statement

The experimental protocol was duly sanctioned by both the Animal Care and Scientific Ethical Committee of Zhejiang University (ZJU202121499).

### Animals, diet composition and experimental design

A total of four thousand newly hatched female chickens (Arbor Acres) were raised under similar husbandry conditions and randomly assigned to five experimental diets for a 42-day feeding period. The chickens were fed a pelletized corn-soybean diet without the use of any medication or vaccination. The dietary treatments consisted of a control diet as the basal diet (CON), and four experimental diets in which increasing amounts of protease were added to the control diet: CON + 100 g/t protease, CON + 200 g/t protease, CON + 300 g/t protease, and CON + 400 g/t protease. The crushed and sieved soybean meal was used as the feed material, and the novel protease was dissolved in water at a ratio of 1:20 (each kg of soybean meal contains 3 g of novel protease). Then the soybean meal powder was mixed with the protease solution and finally pelletized. The novel protease in-feed recovery was determined to be 83.73%. The method used to determine the novel protease was according to the protease preparations (GB/T23527-2009 alkaline protein) [59]. The composition and nutrient content of the corn-soybean basal diets (starter and grower diets) are presented in Additional file 2: Table S1. The protease used in this study was obtained from Bestzyme Bio-Engineering Co., Ltd., specifically Bestzyme ProMax, China.

The experiment was conducted with 8 replicates per treatment, each replicate consisting of 100 birds, each replicate of 10 birds was housed in stainless steel cages (120 cm × 60 cm × 50 cm) with a floor space of 0.72 m^2^ per bird. All birds were fed starter diet when they were 1–21 days old and grower diet when they were 22–42 days old. The birds were provided with ad libitum access to food and water. The temperature in the chicken house was set at 34 °C for the first week and then gradually decreased to 25 °C at a rate of 3 °C per week, and maintained at this temperature until the end of the experiment. The relative humidity in the chicken house was maintained between 45 to 55%, and the birds were maintained on a 18 h light and 6 h dark cycle throughout the experiment.

### Sample collection

On day 39, 40 female birds with moderate weight (one bird per replicate) were randomly selected and weighed after a 12-h period of feed deprivation. Subsequently, the selected 40 birds were humanely euthanized by cervical dislocation. The breast samples were promptly isolated and placed in self-sealing bags, frozen at − 80 °C, and stored for further analysis of IMP, amino acid composition, and fatty acid composition. The remaining sections of the samples were stored at 4 °C in a freezer for the assessment of TVB-N in the breast muscle at 0, 3, 5, 7, and 9 days. Furthermore, samples of cecum contents were swiftly collected and immediately frozen in liquid nitrogen. These samples were then stored at − 80 °C for subsequent analysis of 16S rRNA gene sequences and untargeted metabolomic analysis.

### Determination of IMP and TVB-N

The determination of IMP was performed according to our previously published paper [60]. TVB-N values were measured following the methodology outlined by J Chen, SZ Wang, JY Chen, DZ Chen, SG Deng and B Xu [61], with results expressed in milligrams of TVB-N per 100 g of sample.

### Determination of antioxidant capacity in muscle

To measure the activities of total antioxidant capacity (T-AOC), total superoxide dismutase (T-SOD), glutathione peroxidase (GSH-Px), and catalase (CAT), as well as the content of malondialdehyde (MDA) in the muscles, commercial kits were purchased from Nanjing Jiancheng Institute of Bioengineering (Nanjing, China) and used according to the manufacturer’s instructions.

### Fatty acid and amino acid analysis

Medium and long chain fatty acids were extracted from 200 mg of each sample via blending with 5 ml of a methylene dichloride-methanol solution (2:1, v/v), subsequently employing hexane for the extraction of free fatty acids on two occasions. The resultant supernatant underwent gas chromatography analysis, employing standards to facilitate the identification and quantification of fatty acids. The Varian Star software was employed for chromatogram processing and calculation, with fatty acid content expressed as a percentage of total fatty acids. For short-chain fatty acid analysis, roughly 200 mg of the sample was combined with 50 ml of a 50% phosphoric acid solution. Subsequently, 100 ml of isopropyl ether was introduced to the mixture, which was then homogenized to ensure uniform dispersion prior to analysis.

For the analysis of amino acids, 30 mg of each sample was taken and digested in 10 ml of 6N HCl at 110 °C for 24 h in a hydrolysis tube and then filtered. 0.3 ml of the mixture was extracted into tubes and dried in an oven at 140 °C for 1 h. The dried filtrate samples were mixed and dissolved in 3 ml of sodium citrate buffer solution (pH 2.2) and then analyzed for amino acid content using an amino acid analyzer.

### 16 s rRNA sequencing

Cecum content samples were placed into Eppendorf tubes and preserved at − 80 °C using liquid nitrogen until further analysis. Genomic DNA was extracted from these samples according to the manufacturer's guidelines using the CTAB method. The V3–V4 regions of the 16S rDNA were amplified by PCR using the universal primers 341F-805R. The amplicon libraries were then sequenced, and the quantity and size of the amplicons were assessed using the Agilent 2100 Bioanalyzer (Agilent, USA) and the Library Quantification Kit for Illumina (Kapa Biosciences, Woburn, MA, USA), respectively. The amplicon pools were then subjected to paired-end sequencing on the Illumina NovaSeq PE250 platform, and the sequencing data was analyzed by LC-Bio Technology Co., Ltd. (Zhejiang, China). The initial sequences were merged using FLASH (version 1.2.8), followed by the application of fqtrim (version 0.94) for filtering to obtain high-quality clean tags. Chimeric sequences were then removed using Vsearch software (v2.3.4). Subsequent sequences were dereplicated using DADA2, resulting in a feature table and feature sequence. Alpha diversity and beta diversity were calculated through random normalization of the sequences. Feature abundance was normalized according to the relative abundance of each sample, utilizing the SILVA (version 138) classifier. Alpha diversity was analyzed using four indices: Chao1, Goods coverage, Shannon, and Simpson. Calculations were performed using QIIME2. Beta diversity was also computed with QIIME2 and visualized with the R package. Sequence comparison was done using BLAST, and feature sequences were annotated using the SILVA database for each representative sequence. Other charts were generated using the R package (v3.5.2).

### Untargeted metabolomic analyses

Untargeted metabolomics analyses were performed as described in our previously published methods [62, 63].

### Statistical analysis

Experimental data was analyzed using ANOVA and Tukey's post-hoc test for multiple comparisons in SPSS 20.0 software (IBM-SPSS Inc, United States). The results were reported as means with the Standard Error of the Mean (SEM), and significance was accepted at a value of *P* < 0.05. The GraphPad Prism 8.0.2 software (Monrovia, CA, United States) was used for data visualization and statistical analysis. Correlations among microbiota, metabolites, and slaughter data were determined using Spearman correlations.

### Supplementary Information

**Additional file 1: Fig. S1.** Effects of novel protease feed on growth performance (**A**). Effects of novel protease feed on antioxidant capacity of breast muscle (**B**). ^a−c^ Different lowercase letters indicate significant difference between groups (*P* < 0.05).

**Additional file 2: Table S1. **The composition and nutrient levels of diets for broiler chickens (air dry basis).

## Data Availability

The datasets produced and/or analyzed during the current study are available from the corresponding author on reasonable request.

## Abbreviations

CON: The basal diet
EAA: Essential amino acids
FAA: Flavor amino acids
IMF: Intramuscular fat
IMP: Inosine monophosphate
LDL: Low density lipoprotein
LEfSe: The linear discriminant analysis effect size
MDA: Malondialdehyde
OCFA: Odd-chain saturated fatty acids
PCA: Principal component analysis
PCoA: Principal coordinate analysis
PLS-DA: Partial least squares discriminant analysis
PUFA: Polyunsaturated fatty acids
SCFA: Short chain fatty acids
SEM: Standard error of the mean
SFA: Saturated fatty acids
TAA: Total amino acids
T-AOC: Total antioxidant capacity
TFAC: Total fatty acid content
TP: Total protein
TVB-N: Total volatile basic nitrogen
UFA: Unsaturated fatty acids
VIP: Variable importance in the projection
