# Supplementary figures and images for: Dietary novel alkaline protease from Bacillus licheniformis improves broiler meat nutritional value and modulates intestinal microbiota and metabolites
Source: Anim Microbiome. 2024 Jan 6;6:1. doi: 10.1186/s42523-023-00287-z (PMC10770948; doi:10.1186/s42523-023-00287-z)

## Slide 1
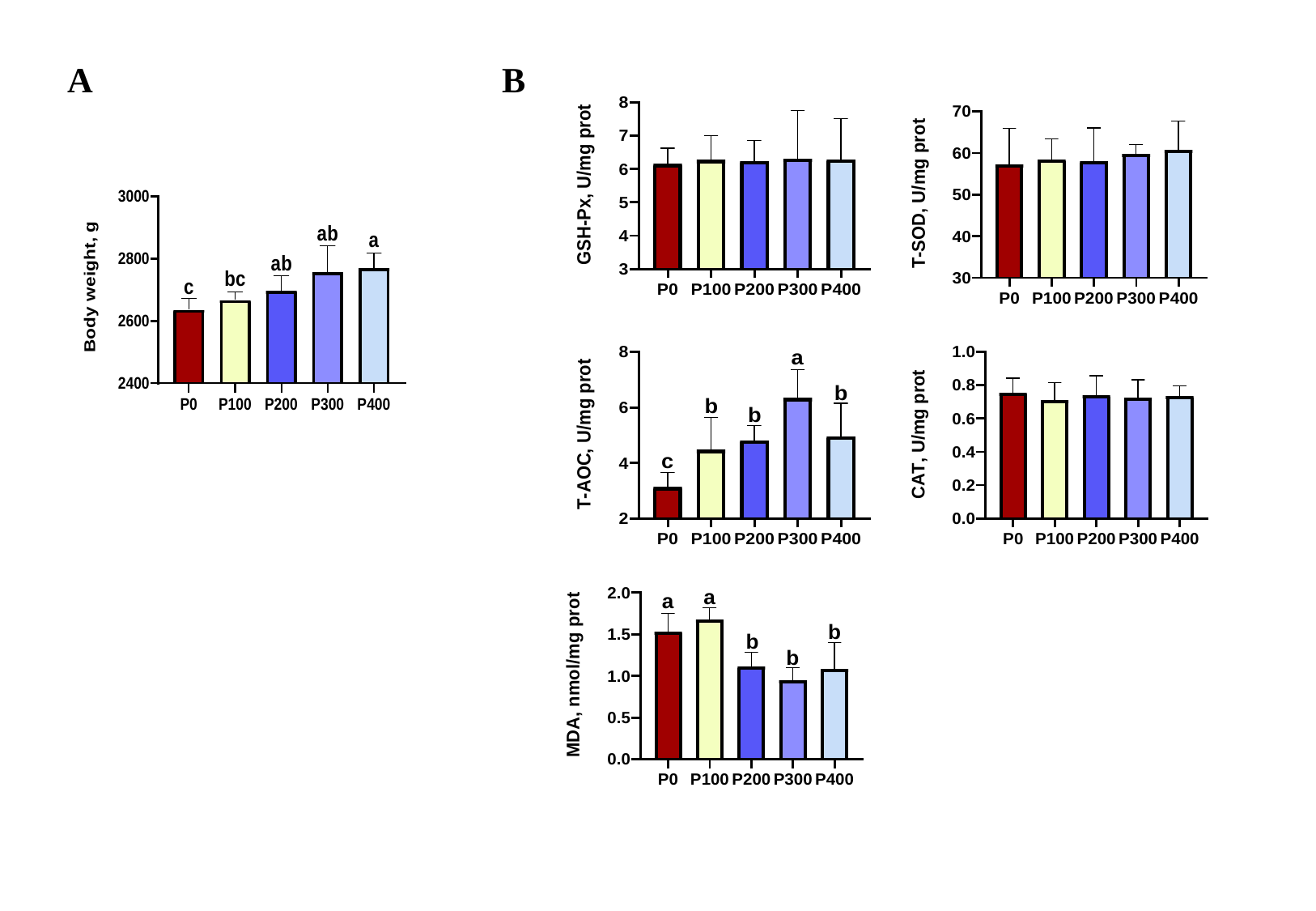

A
B

Supplement: Supplementary file 1 — Additional file 1: Fig. S1. Effects of novel protease feed on growth performance (A). Effects of novel protease feed on antioxidant capacity of breast muscle (B). a−c Different lowercase letters indicate significant difference between groups (P < 0.05). [file 42523_2023_287_MOESM1_ESM.pptx]
