# Supplementary material for: Dietary novel alkaline protease from Bacillus licheniformis improves broiler meat nutritional value and modulates intestinal microbiota and metabolites
Source: Anim Microbiome. 2024 Jan 6;6:1. doi: 10.1186/s42523-023-00287-z (PMC10770948; doi:10.1186/s42523-023-00287-z)
Supplement: Supplementary file 2 — Additional file 2: Table S1. The composition and nutrient levels of diets for broiler chickens (air dry basis). [file 42523_2023_287_MOESM2_ESM.docx]

Table S1. The composition and nutrient levels of diets for broiler chickens (air dry basis).

| **Items** | **Starter phase (1-21 d)** | **Grower phase (22-42 d)** |
| --- | --- | --- |
| *Ingredients, %* |  |  |
| Corn | 58.78 | 61.24 |
| Soybean meal | 25.60 | 23.90 |
| Animal fats | 4.78 | 5.14 |
| Fish meal | 4.00 | 3.00 |
| Soybean oil | 3.00 | 3.00 |
| Lysine | 0.24 | 0.25 |
| Methionine | 0.25 | 0.20 |
| Threonine | 0.05 | 0.05 |
| Dicalcium phosphate | 2.10 | 2.00 |
| Limestone | 0.90 | 0.92 |
| Premix 1 | 0.30 | 0.30 |
| Total | 100.00 | 100.00 |
|  |  |  |
| *Calculated nutrient content* |  |  |
| ME, MJ/kg ^1^ | 13.00 | 13.03 |
| Crude protein, % ^2^ | 20.89 | 20.12 |
| Calcium, % ^3^ | 1.00 | 0.90 |
| Available phosphorus, % ^3^ | 0.55 | 0.50 |
| Methionine, % ^3^ | 0.49 | 0.44 |
| Lysine, % ^2^ | 1.24 | 1.20 |

Premix provided the following per kilogram of diet: vitamin A, 15,000 IU; vitamin D3, 2,000 IU; vitamin E, 30 IU; vitamin B1, 3 mg; vitamin B12, 0.02mg; vitamin B2, 1mg; vitamin K, 0.05 mg; vitaminB6, 0.5 mg; choline chloride, 1 mg; biotin, 0.2 mg; folic acid, 1mg; niacin, 30 mg; pantothenic acid, 15 mg; lysine, 2 mg; methionine, 0.5 mg; threonine, 0.8 mg; Nacl, 2.5 mg; Fe, 70 mg; Zn, 45 mg; Mn, 60 mg; Cu, 10 mg; I, 1.2 mg; Se, 0.4 mg.

^1^ ME based on calculated values.

^2^ Measured in the laboratory.

^3^ Calculated based on D Chen and B Yu [60].
